# Artificial light at night suppresses the day-night cardiovascular variability: evidence from humans and rats

**DOI:** 10.1007/s00424-023-02901-0

**Published:** 2024-01-04

**Authors:** Lubos Molcan, Katarina Babarikova, Diana Cvikova, Natalia Kincelova, Lenka Kubincova, Hana Mauer Sutovska

**Affiliations:** Department of Animal Physiology and Ethology, Faculty of Natural Sciences, Comenius University, Ilkovicova 6, Bratislava, Slovakia

**Keywords:** Artificial light at night, The cardiovascular system, The sympathetic nervous system, Adrenal gland, Diurnal and nocturnal animals

## Abstract

Artificial light at night (ALAN) affects most of the population. Through the retinohypothalamic tract, ALAN modulates the activity of the central circadian oscillator and, consequently, various physiological systems, including the cardiovascular one. We summarised the current knowledge about the effects of ALAN on the cardiovascular system in diurnal and nocturnal animals. Based on published data, ALAN reduces the day-night variability of the blood pressure and heart rate in diurnal and nocturnal animals by increasing the nocturnal values of cardiovascular variables in diurnal animals and decreasing them in nocturnal animals. The effects of ALAN on the cardiovascular system are mainly transmitted through the autonomic nervous system. ALAN is also considered a stress-inducing factor, as glucocorticoid and glucose level changes indicate. Moreover, in nocturnal rats, ALAN increases the pressure response to load. In addition, ALAN induces molecular changes in the heart and blood vessels. Changes in the cardiovascular system significantly depend on the duration of ALAN exposure. To some extent, alterations in physical activity can explain the changes observed in the cardiovascular system after ALAN exposure. Although ALAN acts differently on nocturnal and diurnal animals, we can conclude that both exhibit a weakened circadian coordination among physiological systems, which increases the risk of future cardiovascular complications and reduces the ability to anticipate stress.

## Introduction

Everything cannot happen at once. The coordination of complex biochemical and physiological events in response to a wide range of stimuli from the internal and external environment is crucial to ensure the efficacy of biological functions. Biological processes oscillate with different periods, and the most dominant is the circadian (approximately 24-h) generated by the circadian system (reviewed in [88]). The circadian system comprises the central oscillator (the suprachiasmatic nuclei of the hypothalamus; SCN) and peripheral oscillators. Peripheral oscillators are present in every cell of the organism (reviewed in [75]), and their activity is coordinated directly and indirectly by the central oscillator as well as by various other cues [16]. It is generally assumed that the synchronisation of the peripheral oscillators by SCN occurs primarily through the nervous and endocrine systems (reviewed in [11]). Since SCN has a direct connection with the retina through the retinohypothalamic tract [32], disruption of the regular light–dark regime (quality, intensity, duration and timing) can alter SCN activity and circadian rhythms. Moreover, such alterations are associated with the development and progression of cardiovascular diseases [93].

Regular light input can be disrupted by shift work, night work, jet lag and artificial light at night (ALAN). Compared to other stimuli, ALAN affects the entire population in developed regions [23], including wild animals, and often even before they are born [21]. Significant sources of ALAN are streetlamps, billboards, vehicles and buildings. Whilst natural night-time light exists in the environment, it typically occurs at lower and variable intensities throughout the lunar cycle. For comparison, the intensity of light at night in extreme cases (a supermoon) is up to 0.3 lx, generally up to 0.1 lx [45]. Light intensity varies significantly even in the cities, whereas around streetlamps, it can reach several tens of lux. However, this value highly depends on the light source's distance and location [31]. Nevertheless, evaluating the exact light intensity in human settlements is challenging due to the variability of the Earth's atmosphere and various techniques [42].

The light activates the SCN by direct and indirect neural projections in diurnal and nocturnal animals [40]. Neurotransmitters play a key role in the differential response to light observed in physiological and behavioural expressions in diurnal and nocturnal animals. In diurnal animals, the signal from the subparaventricular nuclei to the paraventricular nuclei is mediated by excitatory glutamatergic neurons. In contrast, in nocturnal animals, this signal is mediated by inhibitory gamma-aminobutyric acid neurons [40].

There are three possible pathways through which the SCN can affect the activity of the cardiovascular system: The first one involves the pineal gland and melatonin release. The second pathway affects the cardiovascular system through the autonomic nervous system. The last one involves the endocrine system [76]. Moreover, light can also have an SCN-independent effect on the cardiovascular system by locomotor activity modulation [72].

In general, light activates the SCN and inhibits melatonin production. Therefore, melatonin levels are high during the dark phase of the day in both diurnal (active during the daytime) and nocturnal (active during the nighttime) animals [4, 55]. The physiological consequences of ALAN are often investigated by measuring changes in melatonin levels [13, 33, 85]. In humans, ALAN decreased melatonin levels in dose-dependent patterns [33]. Also, in diurnal zebra finches [54] and European blackbirds [18], a light intensity-dependent decrease in plasma melatonin was shown, whilst even < 1 lx had a significant effect. A similar decrease in plasma and pineal gland melatonin was also shown in nocturnal male Wistar rats after 2 and 5 weeks of 1–2 lx ALAN [55, 65]. Interestingly, the effects of ALAN can vary depending on whether the animal is in its natural habitat or under controlled experimental conditions. For example, in the case of European perch, under laboratory conditions, already 1 lx during the dark phase significantly reduced the secretion of melatonin [9], in contrast to an experiment in the natural environment, when one month of 15 lx ALAN did not change the melatonin levels in perch [10]. ALAN generally decreases melatonin in both diurnal and nocturnal animals; therefore, its involvement in cardiovascular regulation is questionable (reviewed in: [15]).

On the other hand, other physiological processes, including cardiovascular regulation, differ significantly depending on whether the organism is diurnal or nocturnal [2, 40, 81]. The autonomic nervous system, characterised by a pronounced circadian rhythm, directly influences the heart and blood vessels. The effects of light on sympathetic nerve activity vary according to whether the organism is diurnal or nocturnal and can be either stimulating or inhibiting [78]. In diurnal animals and humans, light stimulates the sympathetic nervous system [78]. Conversely, in nocturnal animals, light inhibits the sympathetic nervous system during the light phase of the day [83]. The activity of the heart and blood vessels is also affected by glucocorticoids [22, 90]. Similar to the autonomic nervous system and catecholamines, glucocorticoids exhibit a significant day-night variability, with a considerable increase in humans in the morning before awakening [95] and in rats at the end of the light (passive, resting) phase of the day [64]. The relevance of glucocorticoids in the context of ALAN exposure is noteworthy for two main reasons. Firstly, ALAN could be perceived as a stress-inducing factor like other circadian disruptions such as shift work [38]. Secondly, glucocorticoids are a hormonal output and feedback signal for the circadian system [79].

Thus, ALAN affects systems that regulate the activity of the cardiovascular system. However, research on ALAN in humans is currently limited, while animal experimental models often involve nocturnal animals such as rats and mice. On the other hand, when diurnal animals are used as animal models, the studies embrace an ecological perspective. However, the response of wildlife to ALAN is complex and influenced by many factors, including migration patterns and predators [17].

Therefore, this work summarises knowledge (until Q1/2023) concerning the impact of ALAN on cardiovascular functioning and determines whether the consequences of ALAN exposure differ between diurnal and nocturnal animals. Within this work, we focus on how ALAN 1) changes blood pressure and heart rate, 2) affects the regulatory mechanisms that are mostly studied in the context of the cardiovascular system, specifically the autonomic nervous system and glucocorticoids, 3) regulates molecular changes in the heart and blood vessels and 4) affects the cardiovascular system via changes in locomotor activity.

## Heart rate and blood pressure

### Diurnal animals

In the human population, exposure to ALAN has been associated with increased susceptibility to cardiometabolic disease, obesity and type 2 diabetes [41, 85, 86]. However, there is currently a lack of adequately controlled, long-term studies that comprehensively assess the effects of ALAN on the cardiovascular system. At a high intensity, ALAN is commonly used in intensive care units. However, this environment is specific, and the impact of ALAN is probably marginal since patients in intensive care units are older, and their vital functions and homeostasis are fundamentally disturbed. Nevertheless, there is an assumption that an increased difference in the light intensity between the light and dark phases can support the circadian variability of cardiovascular parameters, thus accelerate the stabilisation and treatment of patients [43, 44, 68].

Studies with older people in home settings showed that blood pressure and intima-media thickness positively correlated with ALAN intensity, indicating an increased cardiovascular risk. Blood pressure increased especially during light-contaminated nights, and thus, day-night variability decreased [61, 62]. However, it is worth noting that the study was done in an urban population, often exposed to higher noise and stress levels [3]. In middle-aged people (44.2 ± 8.0 years; n = 6,869), who generally have better cardiovascular health compared to the elderly, no relationship was observed between ALAN (self-reported intensity as “darkest”, “middle”, “lightest”) and blood pressure or glomerular filtration. The same study showed that shift work was associated with increased blood pressure and decreased glomerular filtration [94]. In young adults (18–40 years; n = 20), moderate light (100 lx, one night) during sleep increases the night-time heart rate [50]. Similarly, a positive correlation between the intensity of bedroom lights and blood pressure was observed in a cohort study in healthy adults (16–22 years; n = 400), in whom an increase in light intensity by 1 lx corresponded to a rise in systolic blood pressure by 0.55 mmHg, and light intensity > 5 lx was associated with a threefold higher incidence of hypertension [91]. Dim light at night also increases the heart rate in wild diurnal birds. A study conducted on barnacle geese showed that these diurnal birds exhibited an increased heart rate during the dark phase in response to “supermoon” events. These findings indicate that wild diurnal animals react to natural and artificial light fluctuations at night with increased heart rate, and only for a certain period, while ALAN persisted [36, 69].

### Nocturnal animals

In normotensive rats (18 weeks old), which exhibit nocturnal activity and are widely used as an experimental model, ALAN (5 weeks; 1–2 lx) reduced blood pressure and heart rate during the dim light phase, which led to a decrease in the daily variability of blood pressure and heart rate [55]. In spontaneously hypertensive rats (18 weeks old), which are characterised by an increased sympathetic nerve activity [47], ALAN (5 weeks; 1–2 lx) attenuated the age-related increase in blood pressure, leaving a daily heart rate variability unaffected. Moreover, significant increases in blood pressure and heart rate during the transitions between the light and dark phases were lost [74]. ALAN (1–2 lx) had the most pronounced effects on blood pressure and heart rate after two weeks of exposure, and day-night variability was partially restored after five weeks of exposure [55, 82]. In contrast, in rats (28 weeks old) prenatally exposed to hypoxia, a significant decrease in day-night variability of blood pressure and heart rate was not present until 5 weeks of ALAN (1–2 lx) [82]. A similar dampening of the blood pressure rhythms was observed after the shift of the light cycle [71]. The significance of circadian variability depends on the negative feedback loop of clock genes such as *Bmal1* [20]. Thus, in another study, Bmal1 knockout rats exhibited lower blood pressure levels than controls with a normal diurnal rhythm [73].

**Fig. 1:**
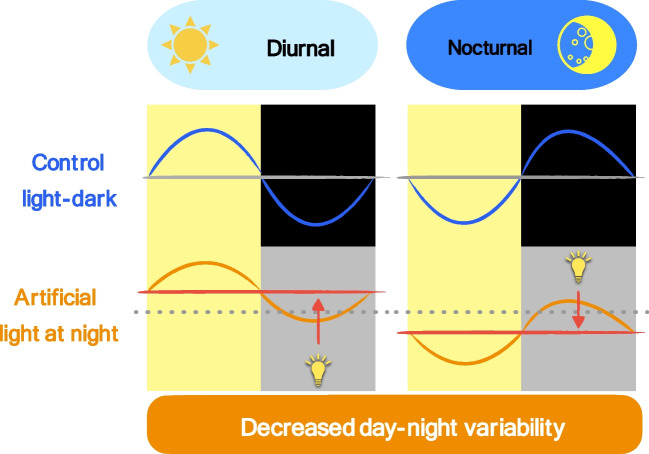
Heart rate and blood pressure vary over 24 h, with higher variability during the control light-dark conditions compared to artificial light at night. Artificial light at night affects daily averages and diminishes the daily variability of heart rate and blood pressure in diurnal and nocturnal animals

In general, we can summarise that ALAN (1) reduces the day-night variability of blood pressure and heart rate in both diurnal and nocturnal animals and (2) increases the daily average of blood pressure and heart rate in diurnal animals, whereas, in nocturnal animals, ALAN decreases the daily average of cardiovascular parameters (Fig. 1).

## The autonomic nervous system

### Diurnal animals

In healthy adults (18–40 years), exposure to moderate light (100 lx) at night increased the low-to-high frequency ratio, indicating a sympathoexcitatory effect of light associated with increased heart rate. The study also observed changes in the macrostructure of sleep, which the authors also related to increased sympathetic activity [50]. However, there is a lack of additional studies investigating the effects of ALAN on heart rate variability and the autonomic nervous system in humans. The available data suggest that exposure to dim light during the night, whether for a limited time or at the end of the night [30, 35], has comparable effects on heart rate variability and the autonomic nervous system to light throughout the night.

### Nocturnal animals

**Fig. 2:**
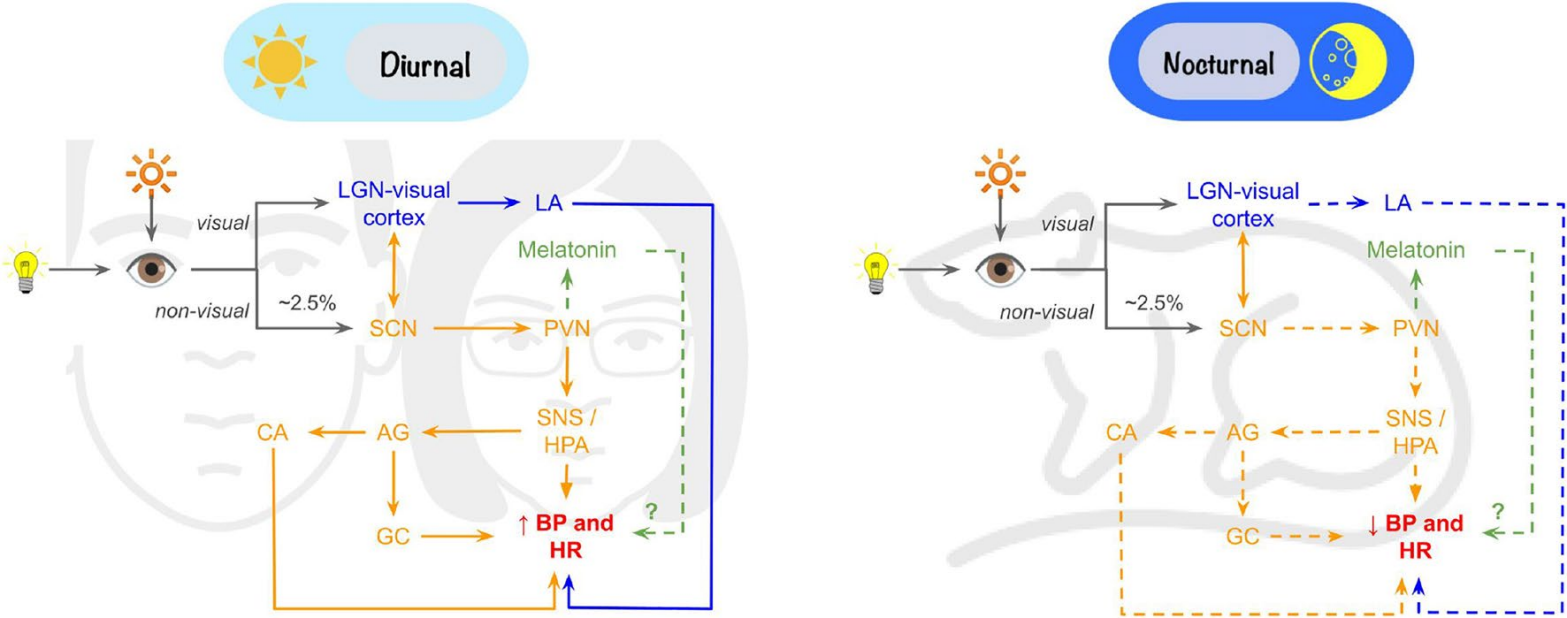
Blood pressure (BP) and heart rate (HR) responses to light in diurnal and nocturnal animals differ, both through direct photic effects and indirectly through non-photic effects via the suprachiasmatic nuclei of the hypothalamus (SCN). AG, adrenal glands; CA, catecholamines; GC, glucocorticoids; HPA, hypothalamic-pituitary-adrenal axis; LA, locomotor activity; LGN, lateral geniculate nucleus; PVN, hypothalamic paraventricular nuclei; SNS, sympathetic nervous system

Studies in spontaneously hypertensive rats [74] and rats exposed prenatally to hypoxia, which experimentally increases sympathetic nerve activity [82], suggest that the sympathetic nervous system is essential in transmitting information from the SCN to the cardiovascular system [83]. The sympathetic nervous system acts on the cardiovascular system through noradrenaline, released from the nerve terminals. At the same time, the sympathetic nervous system stimulates the adrenal medulla to release catecholamines into circulation [39]. In rats exposed to a regular light-dark regime, noradrenaline elicits a higher blood pressure response during the light (passive) phase than the dark (active) phase. However, this phase-dependent response is lost under ALAN conditions, with blood pressure exhibiting a significantly increased response to noradrenaline even during the dark phase [55, 56, 82]. There are several hypotheses for this phenomenon: (1) The first is related to vascular tone, which is significantly controlled by the sympathetic nervous system. An increased vascular tone allows for more significant vasodilation when needed, as shown after methoxamine (an alpha1-agonist) administration [24]. However, if ALAN decreases sympathetic activity, the basal vascular tone is also lower (Fig. 2). Exogenously applied noradrenaline causes vasoconstriction, which is minimally compensated by reflex vasodilation due to the already inhibited sympathetic activity. (2) The more pronounced and prolonged increase in blood pressure after noradrenaline may result from an impaired availability of enzymes responsible for the degradation of catecholamines [34, 55]. However, we did not find any data about the effects of ALAN on the plasma levels of catecholamines or enzymes involved in their turnover. (3) Exposure to ALAN (1–2 lx, 2 weeks) increases the expression of sarco/endoplasmic reticulum Ca^2+^-ATPase type 2 in vascular smooth muscle cells, which may result in increased intracellular calcium storage (Sutovska et al., under review). Consequently, the application of noradrenaline can trigger the release of more calcium into the cytosol, thereby enhancing contractility. Moreover, decreased catecholamine turnover can prolong the pressure response. However, there may be other mechanisms that have not yet been explored or fully investigated. In summary, these findings indicate that the autonomic regulation of the cardiovascular system is one of the crucial pathways through which the central oscillator directly and comprehensively regulates the activity of the heart and blood vessels.

In humans and nocturnal rats, it is likely that ALAN [30, 82], as well as phase shifts of the light-dark regime [57], can alter the sympathovagal balance, which is manifested by increased sympathetic activity in humans and, conversely, decreased sympathetic activity in rats.

## Glucocorticoids

### Diurnal animals

Regarding light pollution, glucocorticoids are given considerable attention, especially considering ALAN as a stressor. Given their influence on cardiovascular function [22, 90], assessing changes in glucocorticoid levels could provide valuable insights into the impact of ALAN on an animal’s cardiovascular system. ALAN effects on glucocorticoid levels are mostly studied in birds [1, 53, 67], fish and frogs, with comparatively fewer studies conducted on humans. However, numerous human studies have been conducted on shift work, revealing disrupted daily cortisol rhythms with no significant changes in average cortisol levels [58, 59]. Interestingly, diurnal animals do not show a uniform hypothalamic-pituitary-adrenal axis response to ALAN. In healthy men (23.4 ± 1.5 years), ALAN (< 5 lx; 2 days; n = 20 [14]) as well as bright light at night (around 9,500 lx; 1 day; 24.6 ± 5.1 years; n = 21 [70]), have been shown to reduce plasma cortisol levels. However, acute one-day intermittent bright light increased plasma cortisol [70]. Similarly to humans, adult cane toads exhibited decreased salivary corticosterone after 12 days of 0.04 lx and 5 lx ALAN [77].

On the other hand, in Nile grass rats, ALAN (three weeks) elevated daytime corticosterone levels [26]. Also, diurnal songbirds (male baya weavers) exhibited increased plasma corticosterone levels following acute exposure to ALAN (1 week). After chronic exposure (4 weeks), the observed increase in corticosterone levels was diminished, indicating a specific type of habituation to ALAN [92].

In wild fish, neither acute (48 and 80 lx), long-term (around 15 lx), nor pre-hatch ALAN (1–8 lx) had a significant effect on cortisol levels [9, 60, 84]. However, glucose levels (stress indicator) were significantly increased after ALAN (5–15 lx during the dawn period; 9 days; laboratory conditions [37]), as well as one-day exposure to continuous (48 lx) or intermittent (80 lx) light at night [84]. The authors assumed that since the stress was only acute, cortisol may have already returned to normal levels at the time of measurement [84], as shown previously [49]. Notably, decreased locomotor activity was observed in some fish during ALAN exposure, leading the authors to assume that ALAN-resistant animals change their behaviour rather than an endocrine stress response [52].

### Nocturnal animals

**Fig. 3:**
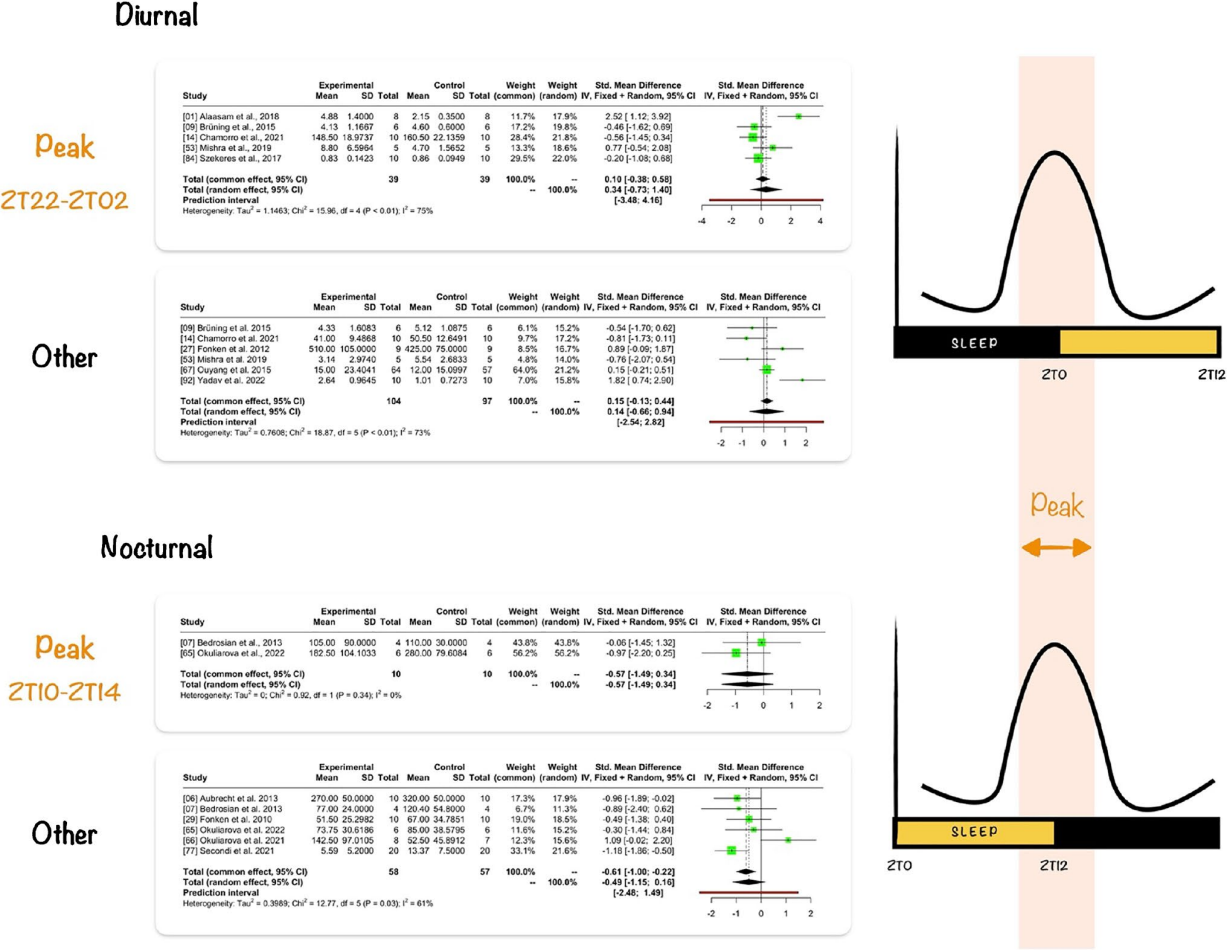
Meta-analysis with continuous outcome of glucocorticoids in diurnal and nocturnal animals. ZT0 represents the beginning of the light phase; CI, confidence interval; SD, standard deviation

Unlike diurnal animals, nocturnal animals exhibit peak corticosterone levels at the end of the light (passive) phase of the day [64]. Under laboratory conditions, various effects of ALAN on corticosterone levels have been shown in rodents (Fig. 3):

1. No changes in serum corticosterone levels and hippocampal glucocorticoid receptor expression were observed after ALAN (5 lx; 3 days) in female and male Swiss Webster mice during both the light and the dark phases of the day [89]. Even longer exposure to ALAN (5 lx; 3–7 weeks) did not affect the corticosterone levels in mice in the light [6, 29] and the dark [29] phases of the day.
2. Suppression of the diurnal rhythm of serum cortisol after ALAN (5 lx; 1 week) was observed in adult female Siberian hamsters [7]. In rats, 2 weeks of 2 lx ALAN suppressed and shifted corticosterone rhythm [65].
3. Increased plasma corticosterone levels were observed in C57BL/6J mice (5 lx; 4 weeks) [46], male Nile grass rats in the light phase (5 lx; 2 weeks) [27], and male Wistar rats in the first half of the light phase (ZT03 – ZT06; 2 lx; 2 and 5 weeks) [66].

Given the diverse findings across publications on glucocorticoids, we conducted a meta-analysis of 20 publications investigating glucocorticoids in relation to ALAN. We excluded six studies due to incomplete data or information about the time of data collection. The meta-analysis included 14 studies, six devoted to nocturnal and eight to diurnal animals. We only analysed data where the light was defined other than constant light. The median light intensity in the papers was 5 lx (min: 0.04 lx, max: 400 lx). We normalised the time in the publications to the beginning of the light phase (ZT0). Based on the ZT, we divided the data for the meta-analysis according to diurnal and nocturnal animals, and we then split the data sets according to the expected glucocorticoid peak time and the rest of the day, when the maximum for glucocorticoids is not expected. We thus created four data sets: 1) diurnal animals ZT22–ZT02 (expected peak), 2) diurnal animals ZT02–ZT22, 3) nocturnal animals ZT10–ZT14 (expected peak) and 4) nocturnal animals ZT14–ZT10. In diurnal and nocturnal animals, glucocorticoid levels increase at the end of the passive phase [51].

Since different studies employed different measurement techniques and units for glucocorticoid quantification, we converted all units to ng, if possible. However, glucocorticoids were sometimes expressed per litter/kg, gram, and ml. Furthermore, we used standardised mean differences to combine continuous data using fixed- and random-effect models (Fig. 3). We observed significant variation in study outcomes between studies (heterogeneity) in three of four data sets (Fig. 3). Insignificant heterogeneity was in the data sets of nocturnal animals that measured glucocorticoids during the expected peak (ZT10–ZT14), probably due to the limited number of studies (n = 2). These papers showed that ALAN reduces glucocorticoid levels during the expected rise. The measurement of glucocorticoids in the remaining three data sets involving nocturnal and diurnal animals showed mixed results, with some showing an increase, some a decrease, and others reporting no changes in glucocorticoid levels after ALAN (Fig. 3). These differences between studies may be attributed to shifts in the timing of glucocorticoid peaks [65]. The magnitude of glucocorticoid shift could depend on the duration of ALAN exposure. Therefore, we tried to minimise the shift effect with a 4-h peak interval. However, this interval might not be sufficient if ALAN exposure is prolonged. On the other hand, since not all papers had precisely defined experimental conditions, there could be different possibilities of significant heterogeneity. In future studies, the measurement of corticosterone and other parameters with apparent circadian variability requires monitoring throughout a 24-h cycle to arrive at a clear conclusion since ALAN likely changes the amplitude and acrophase of the measured parameters.

To sum up, the overall impact of ALAN on glucocorticoids is not uniform within diurnal or nocturnal species. Studies on humans and frogs show a decrease in glucocorticoid levels following ALAN exposure, whereas fish appear resistant to ALAN despite some displaying increased glucose levels. On the other hand, diurnal songbirds and nocturnal rodents exhibit increased glucocorticoid levels after ALAN exposure. Additionally, it's probably important to consider an animal's behavioural strategies beyond its diurnal or nocturnal nature. ALAN may play a role in altering predator-prey dynamics, thus contributing to the stress experienced by the animal. Heterogeneity in study outcomes highlights the need for standardised experimental protocols and continuous monitoring throughout a 24-h cycle to draw more definitive conclusions.

## Molecular changes in the heart and vessels

Catecholamines and glucocorticoids significantly affect the heart and blood vessels, possibly modulating the expression of proteins within tissues.

### Diurnal animals

In humans, the effects of ALAN on cardiovascular morphology have only been studied on carotid artery intima-media thickness. Carotid artery intima-media thickness is generally used as a marker of subclinical atherosclerosis burden [62, 63]. The increase in intima-media thickness was related to ALAN intensity. The authors of the study estimate that an increase of only 0.083 mm (95% CI, 0.037–0.129) in the maximum carotid intima-media thickness due to ALAN can lead to a 10.0% (95% CI, 3.4–16.4) increase in the probability of myocardial infarction and an 11.6% (95% CI, 4.0–19.2) increase in the probability of ischaemic stroke. However, the authors only point to the effects and do not address the mechanisms [62, 63]. In addition, these results cannot be generalised to the whole population, as the effects of ALAN were studied in older people in an urban home environment, where additive factors may have interacted simultaneously.

In diurnal zebra finches, only males exposed to ALAN (1.5 lx; 10 days) exhibited increased cardiac weight by one-quarter. On the other hand, the authors did not observe left ventricular fibrosis, changes in the expression of transforming growth factor beta, or alterations in the phosphorylation of extracellular signal-regulated kinases and c-Jun N-terminal kinases. These findings suggest that the cardiac hypertrophy was physiological rather than pathological, probably caused by the increased locomotor activity observed [2].

### Nocturnal animals

In mice and rats, ALAN after four (5 lx) and five weeks (1–2 lx) did not affect heart weight, respectively [25, 81]. However, in normotensive and hypertensive male rats, short-term exposure to ALAN (1–2 lx; 2 weeks) resulted in a significantly decreased expression of angiotensin II receptor type 1 in the heart [81] and an increased expression of eNOS in the thoracic aorta, whereas the vascular expression of endothelin-1 remained unchanged [55]. Prolonged exposure to ALAN (1–2 lx; 5 weeks) resulted in the decreased cardiac expression of the sarco/endoplasmic reticulum Ca^2+^-ATPase and endothelin-1 [81]. However, in the thoracic aorta, eNOS and endothelin-1 expression remained unchanged [55].

In summary, ALAN exhibits distinct impacts on diurnal and nocturnal animals. Nocturnal animals exposed to ALAN typically exhibit vasodilation, along with a decrease in heart rate and contractility. Conversely, diurnal animals respond to ALAN with an increased heart rate, which may reflect increased locomotor activity.

## Locomotor activity

Locomotor activity is one of the basic parameters that are often used to describe the activity of the SCN (reviewed in [48]). Furthermore, it plays a significant role in modulating the activity of the cardiovascular system (Fig. 2). However, especially in nocturnal animals, light directly affects animal behaviour and locomotion (masking effect, reviewed in [88]), thereby making the influence of the SCN less pronounced [5].

### Diurnal animals

In male great tits, exposure to ALAN (0.5 lx; 1.5 lx and 5 lx; 22 days) significantly influenced their daily activity. The group exposed to 5 lx ALAN displayed increased activity 6–7 h before daylight, with up to 40% of their daily activity occurring at night. In comparison, in the control group, nocturnal activity accounted for 1% of the total daily activity. In the 0.5-lx group, 11% of the activity occurred at night and in the 1.5-lx group, it was 14% [19]. In wild-caught male songbird baya weaver, exposure to ALAN (5 lx; 4 weeks) significantly increased night-time activity, with the most significant behavioural and physiological changes being observed during the first week of ALAN. However, during the fourth week, the birds adapted to ALAN, and their behaviour and physiology returned to almost the original state [92].

### Nocturnal animals

Wild wood mice are active mainly during darkness. When they were exposed to ALAN (6 weeks, 8 lx at ground level), a decrease in their locomotor activity during the dark phase was observed, which the authors justified as a masking effect of the fear of the predator [80]. Hiding and a preference for darker places have also been shown in the beach mouse, which naturally prefers to collect seeds in areas without artificial light [8]. Hiding from a predator in the wild is well-known and has a feedback effect on the predator's behaviour. For example, the activity of bats increased during ALAN exposure because the light attracted more insects [80]. An altered natural photoperiod significantly affects the behaviour of cane toads, which are predominantly nocturnal (their physical activity negatively correlates with the intensity of the moonlight). In another study, ALAN (5 lx, 12 days) delayed their nocturnal activity by 4.5 h compared to the control group, whereas the number of movements recorded during nocturnal activity was reduced by a fifth due to ALAN. Individuals exposed to ALAN shifted from crepuscular (active primarily during the twilight period) to a more uniform nocturnal activity [77]. A decrease in activity by almost 60% and 75%, depending on ALAN intensity (5 lx vs 20 lx; 10 days), was also observed in male common toads. In addition, ALAN modified the time spent in activity only during the nocturnal period [87].

Conversely, in some studies, ALAN had no effects on physical activity. For example, in adult female Siberian hamsters (crepuscular), ALAN (5 lx; one week) did not alter locomotor activity [7]. Similarly, in male Swiss-Webster mice (5 lx; 8 weeks) and diurnal male Nile grass rats (5 lx; 3 weeks), ALAN did not affect locomotor activity [27, 29]. Moreover, ALAN did not affect total daily wheel running in Swiss-Webster mice, but some animals became arrhythmic [28].

Overall, ALAN exposure can lead to alterations in the locomotor activity in both diurnal and nocturnal animals. Since blood pressure and heart rate depend on locomotor activity, its temporal pattern is important from the point of circadian and SCN-independent regulation of the cardiovascular system (Fig. 2). In nocturnal animals, locomotor activity is reduced if light is present during the dark phase likely due to positive masking. On the other hand, diurnal animals are less sensitive to ALAN, but their locomotor activity may increase during the dark phase at the expense of daytime activity [2, 55]. Moreover, the variability of the cardiovascular system and locomotor activity is modified by food intake, another strong synchronising stimulus [12]. In the context of food intake and masking effects, restoring the day-night variability of blood pressure, heart rate and locomotor activity after more weeks of ALAN is not surprising.

## Limitations

The literature regarding the effects of ALAN on the cardiovascular system, mainly at the molecular level, is limited. We did not find work that addresses the impact of intermittent (over several days) ALAN. If we assume that there are time-dependent changes in the cardiovascular system, intermittent ALAN can significantly affect cardiovascular health. However, respective studies mainly examined the impacts of short-term ALAN exposure, lacking data concerning the effects of chronic ALAN exposure. Furthermore, we did not find data from experimental pathophysiological models exposed to ALAN, such as a failing heart or cardio-renal complications, that would mimic older adults exposed to ALAN. Additionally, there is insufficient evidence of a relationship between ALAN intensity and cardiovascular changes.

## Conclusions

There is substantial evidence that ALAN reduces the day-night variability of cardiovascular parameters in both diurnal and nocturnal animals, indicating a weakened circadian coordination among physiological systems, a worsened predictability of the cardiovascular load and future cardiovascular complications. Short-term ALAN exposure increases the daily averages of blood pressure and heart rate in diurnal animals, whereas, in nocturnal animals, the daily averages of cardiovascular parameters decrease. Based on limited data, the daily variability of cardiovascular parameters seems to gradually restore after several weeks of ALAN compared to SCN and melatonin levels. The effects of ALAN are transmitted from the SCN, most likely through the autonomic nervous and endocrine (catecholamines and glucocorticoids) systems, whereas the role of melatonin is unlikely. To some extent, the changes observed in the cardiovascular system after ALAN exposure can be explained by alterations in locomotor activity, which changes differently during ALAN in diurnal and nocturnal animals.

## Data Availability

Data will be made available on request.
